# Ratiometric Detection of Zn^2+^ Using DNAzyme-Based Bioluminescence Resonance Energy Transfer Sensors

**DOI:** 10.3390/chemistry5030119

**Published:** 2023-08-08

**Authors:** Yuting Wu, Whitney Lewis, Jing Luen Wai, Mengyi Xiong, Jiao Zheng, Zhenglin Yang, Chloe Gordon, Ying Lu, Siu Yee New, Xiao-Bing Zhang, Yi Lu

**Affiliations:** 1 Department of Chemistry, University of Texas at Austin, Austin, TX 78712, USA;; 2 Department of Chemistry, University of Illinois at Urbana-Champaign, Urbana, IL 61801,; 3 School of Pharmacy, Faculty of Science and Engineering, University of No0ingham Malaysia, Semenyih, Selangor 43500, Malaysia;; 4 Molecular Science and Biomedicine Laboratory, State Key Laboratory of Chemo/Biosensing and Chemometrics, College of Chemistry and Chemical Engineering, Hunan University, Changsha, Hunan 410082, China;; 5 Department of Biochemistry, University of Illinois at Urbana-Champaign, Urbana, IL 61801, USA;

**Keywords:** DNAzyme, bioluminescence resonance energy transfer (BRET), metal detection, DNA machines for biosensing

## Abstract

While fluorescent sensors have been developed for monitoring metal ions in health and diseases, they are limited by the requirement of an excitation light source that can lead to photobleaching and a high autofluorescence background. To address these issues, bioluminescence resonance energy transfer (BRET)-based protein or small molecule sensors have been developed; however, most of them are not highly selective nor generalizable to different metal ions. Taking advantage of the high selectivity and generalizability of DNAzymes, we report herein DNAzyme-based ratiometric sensors for Zn^2+^ based on BRET. The 8–17 DNAzyme was labeled with luciferase and Cy3. The proximity between luciferase and Cy3 permiQed BRET when coelenterazine, the substrate for luciferase, was introduced. Adding samples containing Zn^2+^ resulted in a cleavage of the substrate strand, causing dehybridization of the DNAzyme construct, thus increasing the distance between Cy3 and luciferase and changing the BRET signals. Using these sensors, we detected Zn^2+^ in serum samples and achieved Zn^2+^ detection with a smartphone camera. Moreover, since the BRET pair is not the component that determines the selectivity of the sensors, this sensing platform has the potential to be adapted for the detection of other metal ions with other metal-dependent DNAzymes.

## 1 Introduction

Metal ions are involved in many biological processes from signaling to catalysis [1–3]. To perform these functions, the concentrations of metal ions are tightly controlled, and any abnormal changes can bring detrimental consequences to human health. For example, zinc deficiency is related to growth failure and variations of Zn^2+^ concentrations have been linked to diseases, such as cancer, diabetes, inflammation, and infection [4–9]. Therefore, Zn^2+^ may serve as a potential biomarker for monitoring the health status of patients [10–13]. Moreover, Zn^2+^ could also be taken orally and has shown benefits in preventing or treating those diseases and improves mortality and modality in patients [14–19]. However, an overdose of zinc supplements could cause zinc poisoning and side effects including nausea, vomiting, and epigastric pain [20,21]. Since levels of serum Zn^2+^ reflect the zinc levels in critically ill patients, there is a need to detect Zn^2+^ in serum in a timely manner [22,23]. Therefore, developing point-of-care (POC) sensors to monitor concentrations of metals like Zn^2+^ in serum can not only help with monitoring health conditions, but also with managing the metal-based drug treatments of patients [24–27].

Currently, metal ion detection mostly depends on spectrometry technologies, including inductively coupled plasma mass spectrometry (ICP-MS) [28–30]. These techniques achieved zinc detection with a detection limit of 0.1 ng/L [31] and a sensitivity of 10^−7^ unit on mass when differentiating from other metal ions [32]; however, they require sophisticated instruments and well-trained technicians, thus are not suitable for POC detection, especially when resources are limited (see Table S1 for a comparison among the existing systems). To address these issues, many fluorescence-based sensors were developed including the commercialized Zn^2+^ assay kits. However, some of these Zn^2+^ indicators also bind other metal ions or chemicals [33–38]. For example, a commonly used Zn^2+^ indicator is (5-bromo-2-pyridylazo)-5-diethylaminophenol (5-Br-PADAP), which shows colorimetric signal when interacting with Zn^2+^ [33–37]. However, this indicator is also used for detecting Cu^2+^ by interacting with Cu^2+^ and generating a colorimetric complex Cu(II)-2-(5bromo-2-pyridylazo)-5-diethylaminophenol. The complex has an overlapped absorption spectrum with zinc-2-(5-bromo-2-pyridylazo)-5-diethylaminophenol, which displays only a small shift in the absorption peaks from 558 nm for Cu^2+^ to 554 nm for Zn^2+^. This overlapped absorption spectrum limited the selectivity of the 5-Br-PADAP [39,40]. Recent developments of a small molecule sensor showed promising selective detection of Zn^2+^ with a detection limit of 2.36 × 10^−8^ M, but it had a small cytotoxicity and was only applied to *E. coli* cells [41].

In comparison to small molecule sensors, DNAzyme-based sensors show promise in sensing a wide variety of metal ions with high selectivity [42–52]. DNAzymes are singlestranded DNA molecules that exhibit enzymatic activities in the presence of their specific target, such as metal ions. Since identified in the early 1990s, many metal-specific DNAzymes have been identified through in vitro selection from a large DNA library pool of up to 10^15^ different sequences [53–55]. They have been converted into metal fluorescent sensors using either catalytic beacon [56–65] or fluorescence resonance energy transfer designs [66–68]. Both sensing strategies take advantage of the energy transfer between the fluorescence donor and acceptors that are conjugated to different DNA strands of the DNAzyme. The distances between DNA strands are associated with the efficiency of the energy transfer and thus can convert the metal dependent DNAzyme cleavage and subsequent strands dissociation to the changes in the efficiency in energy transfer and fluorescence intensities. A primary example of the DNAzyme sensors is the 8–17 DNAzyme [44,69]. This DNAzyme has been applied in Zn^2+^ detection in a wide range of biological samples including serum [70], cells [71–75], zebrafish [76], and mice [77].

While fluorescent sensors are powerful in sensing and imaging metal ions, a drawback for applying such sensors in POC detection is that they require laser excitation for the donor fluorophore. This not only requires specific equipment, but also results in a high autofluorescence background and photobleaching due to the high-energy laser excitation. To overcome this limitation, chemiluminescence that can generate light emission without laser excitation can be used as a signal readout or exploited to excite the compatible acceptor fluorophores. For example, the oxidation of luminol by hemin/G-quadruplex/H_2_O_2_ generates chemiluminescence and is adapted for sensing applications, which includes the combination between DNAzyme and G-quadruplex for metal sensing [78–84]. This system eliminated the need for an excitation light source and also served as the light donor to excite an adjacent fluorophore and achieved resonance energy transfer (CRET), which enabled rapid and portable metal detection with a DNAzyme sensor and smartphone camera [85]. However, due to the toxicity and strong oxidative ability of H_2_O_2_, the CRET sensor can be difficult to apply in various biological samples. To address these issues, we resort to bioluminescence resonance energy transfer (BRET), which relies on a light-emitting donor enzyme to excite an adjacent fluorophore [86–89]. Like CRET, BRET does not require an external excitation source, thus could reduce the autofluorescence and photobleaching tendency. Different from CRET, the donor of BRET is based on bioluminescence, such as luciferase, which is highly compatible with biological samples. Moreover, BRET can also be generated in a time-controlled manner because the bioluminescent enzyme only emits light when the corresponding substrate is provided. Therefore, BRET has very low background interference and minimal photobleaching, as energy from the donor enzyme is far less powerful than that of excitation light sources such as xenon lamps or lasers.

**Figure 1.**
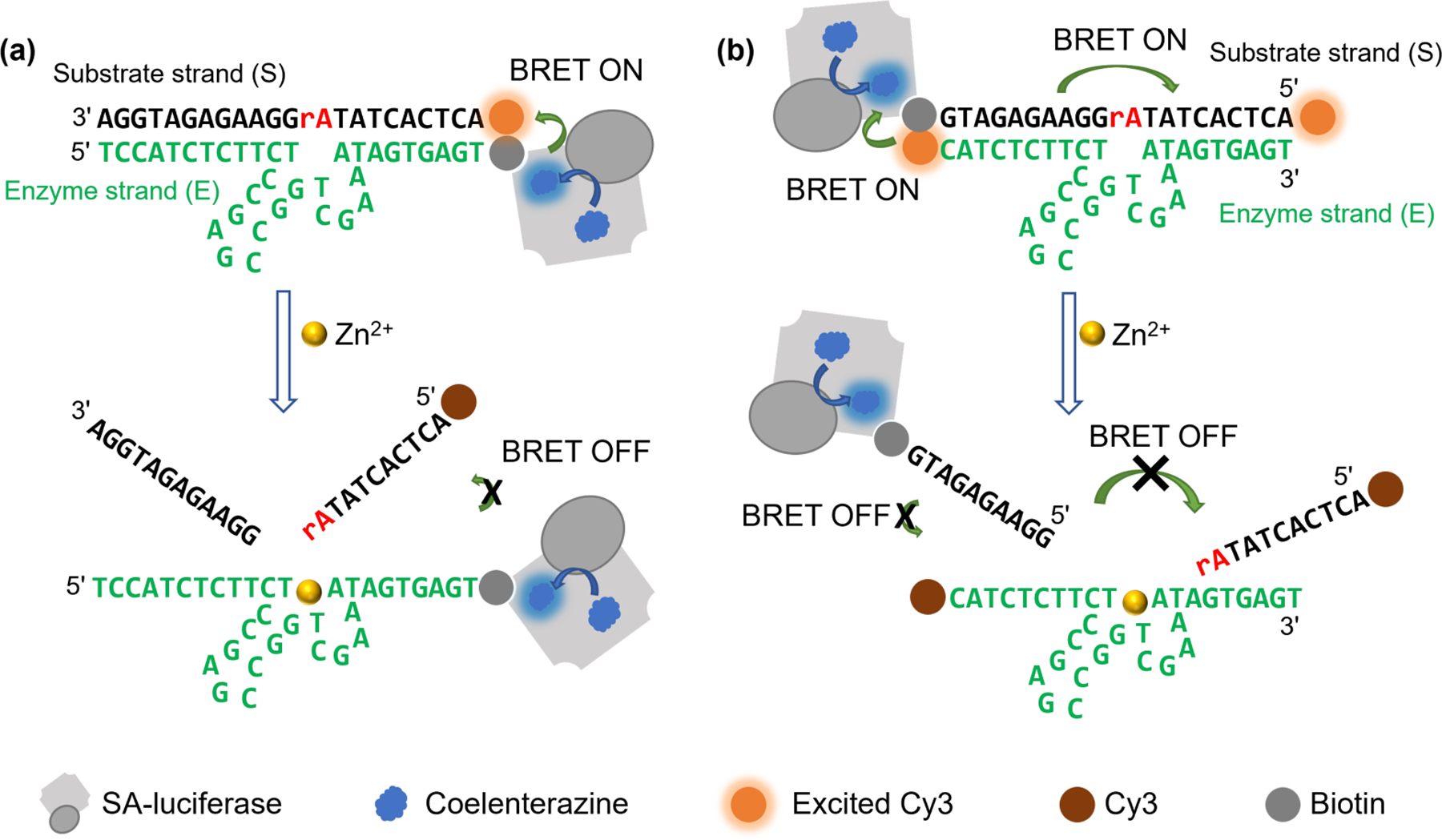
Scheme of the DNAzyme-based BRET sensors. (**a**) A BRET sensor with a single Cy3 acceptor; (**b**) a BRET sensor with two Cy3 acceptors. In both designs, the substrate strand (S) of the 8–17 DNAzyme is hybridized with the enzyme strand (E), which brings the luciferase close to Cy3, resulting in BRET when coelenterazine, the substrate of luciferase, is added. In the presence of Zn^2+^, the S strand is cleaved into two halves, which have lower melting temperatures (T_M_) between the cleaved S strand product and E strand. Due to the change in T_M_, the cleaved fragments dissociate from E strand, effectively distancing Cy3 from luciferase and subsequently inhibiting BRET.

Although BRET-based Zn^2+^ sensors were developed and applied in cellular imaging previously, they were based on the interaction between Zn^2+^ and carbonic anhydrase or cysteine(s)/histidine(s), which were not selective, showing activity towards a few metal ions other than Zn^2+^ [90–92]. Taking advantage of the features of BRET and the selectivity of DNAzyme [44] over the zinc-binding proteins, we report herein the development and optimization of DNAzyme-based BRET sensors (Figure 1) that enable ratiometric detection of Zn^2+^ by utilizing the luminescence as an internal control. With these sensors, we detected and quantified Zn^2+^. In addition, with a smartphone camera, we were able to achieve fast semi-quantitative detection of Zn^2+^ in fetal bovine serum and human serum. The combination of the BRET-based sensor, which does not require sophisticated equipment and lasers that may generate high autofluorescence and photobleaching, with the DNAzyme sensors, which has high selectivity and generalizability for detecting metal ions, advanced the field of bioanalytical chemistry. Moreover, since luciferase and fluorophores are not associated with the selectivity of the DNAzyme, this system has the potential to be used for detecting other analytes by utilizing different DNAzymes.

## 2. Materials and Methods

### 2.1. Instrumentation

The Cary 8454 UV-Vis spectrophotometer was used to obtain absorbance spectra. A FluoroMax-P fluorometer (HORIBA Jobin Yvon Inc., Edison, NJ, USA) and ISS ChronosDFD fluorometer (ISS Inc., Champaign, IL, USA) were used for the luminescent spectra measurements. When working with the FluoroMax-P fluorometer, the instrument was set to have excitation and emission slits of 0 and 20 nm, respectively, to eliminate potential influence from intrinsic excitation light from the instrument itself while maximizing the luminescent signal from the sample. When working with the ISS fluorometer, the instrument was set to have emission slits of 20 nm and a physical shuler was applied for eliminating excitation light from the instrument.

### 2.2. Chemicals

All chemicals were obtained from Sigma-Aldrich and were of analytical grade and used without further purification. A phosphate-buffered saline (PBS, pH 7.5) was prepared by diluting a 10X stock buffer with its pH calibrated using a pH meter. For testing the BRET sensor with single acceptor, streptavidin-Lucia (100 μg/mL, 37 kDa) and its coelenterazine-based luminescence assay reagents Quanti-Luc solution were obtained from InvivoGen and were reconstituted as recommended by the supplier. Streptavidin–Renilla luciferase fusion protein from Raybiotech (500 μg/mL, 54 kDa) was used for testing the BRET sensors with dual Cy3 acceptors due to the Streptavidin-Lucia being discontinued. Deionized water (DI H_2_O) (18.2 MΩ) was used throughout the experiments. Coelenterazine solution for streptavidin–Renilla was purchased from GoldBio (Catalog # CZ). A total of 5 mg coelenterazine was dissolved in 1 mL of acidified methanol (added 50 μL of concentrated HCl to 10 mL methanol) to make stock solution. Small aliquots were stored in a freezer to avoid the instability of the coelenterazine substrate. Fresh aliquots were then used each time without freezing and thawing cycles. Right before each use, 500 μM coelenterazine solution was made by mixing 3153.3 μL Dulbecco′s phosphate-buffered saline (DPBS) with 846.6 μL of the fresh-thawed stock solution. DPBS buffer (pH 7.4) contains 137.9 mM NaCl, 1.47 mM KH_2_PO_4_, 2.67 mM KCl, and 8.09 mM Na_2_HPO_4_, and was bought from Thermo Scientific (Catalog# J67802.K2). Human serum and fetal bovine serum (FBS) were bought from Sigma-Aldrich, heat inactivated and sterile-filtered.

### 2.3. DNA Sequences

DNA and DNA–RNA chimeric oligonucleotides were synthesized by Integrated DNA Technologies, Inc. (Table S2). Fluorophore and quencher labelled sequences were purified by HPLC while other sequences were purified through standard desalting method. All oligonucleotides were reconstituted in DI H_2_O and stored in −20 °C freezer as recommended by manufacturer and used without further purification. UV-Vis spectra were taken to calibrate the concentration of all oligonucleotide-containing solutions prior to usage.

### 2.4. BRET Assays

Both DNAzyme enzyme strand (E) and substrate strand (S) were first heat treated at 95 °C for 5 minutes and slowly cooled to ensure adequate hybridization. The resulting DNA probe (E and S complex) was then mixed with equal volume of analyte solution and subsequently incubated at room temperature to initiate the DNAzyme activity for 30 minutes. After that, an equal volume of luciferase was added to conjugate the biotinylated DNA probe. A total of 50 μL of coelenterazine substrate solution was added right before fluorescence measurement to minimize signal degradation. The total volume of the final sensing solution was 250 μL. The assay was optimized in terms of cool-down techniques, E:S ratio, and DNAzyme probe:streptavidin–luciferase (SA–luciferase) ratio.

For sensitivity, different concentrations of zinc-containing analyte solutions were reacted with the DNA probe. The final concentrations of DNA probe and SA–luciferase were both fixed at 10 nM. The concentrations of working zinc-containing analyte solutions were 0.5, 1, 3, 8, 12, 16, 25, 50, and 100 μM. Since we will need to match the Zn^2+^ concentration with the initial concentration in serum sample for a clinical test, we simplified the conversion by fixing the volume of the sample as 50 μL and used the zinc concentration in the analyte solution instead of final 250 μL BRET system for all the calibration curves, all the Zn^2+^-related reactions, and all the concentrations mentioned in this paper. Thus, the zinc concentration can be directly understood as the concentration in real samples.

For selectivity, various metal ions (Pb^2+^, Mg^2+^, Ba^2+^, Ca^2+^, K^+^, Na^+^, and Li^+^) were used to challenge the sensor and the performances were compared with Zn^2+^ ions. The working concentrations of the metal ions were fixed at 25 μM to obtain noticeable signal differences.

For sensing Zn^2+^ in 1/2 diluted serum, a 120 μL mixture of DNAzyme probe was added into 40 μL serum, which was preloaded with different concentrations of Zn^2+^ or 250 mM EDTA. For sensing undiluted FBS, an 80 μL mixture of DNAzyme probe, containing the same amount but more concentrated probes, was added into 80 μL FBS containing different concentrations of Zn^2+^. For all the BRET experiments, fluorescence spectra were recorded at 30 seconds after adding in 40 μL coelenterazine solution to the system.

### 2.5. BRET POC Imaging

The same BRET assay system was prepared and set up in a white colored 96 well plate. After Zn^2+^ incubation, equal amount of coelenterazine at 40 μL was added to each well using muti-channel pipele. Right after adding in the coelenterazine, the plate was placed into a black box with only a small opening for the camera. The picture was taken within a black box and inside of a dark room with professional imaging mode of a Samsung cell phone (Galaxy S22 Ultra, 30 seconds exposure time and ISO 640). To avoid small movements that could result in a blurred picture due to the long exposure time, the smartphone was placed on the black box without being held by hands when taking the picture.

### 2.6. ICP-MS

The human serum samples we used for validating BRET sensors were tested with ICP-MS to quantify and validate the metal amount. Human serum samples were digested and diluted 250-fold with 2% HNO_3_ to make sure the final total dissolved salt in the system was under 200 ppm. Additionally, to reflect the buffer conditions that we used for the POC imaging and BRET sensor detections, we prepared 10% serum sample in PBS without Ca^2+^ and Mg^2+^ and spiked with different concentrations of ZnCl2. The 10% serum samples were digested and further diluted 250-fold by 2% HNO_3_. Digested serum samples were centrifuged, and the supernatant was sent to ICP-MS facility lab at the Jackson School of Geosciences at the University of Texas at Austin for detection. Blank 2% HNO_3_ buffer were tested between samples, and three independent quality controls with three replicates each were tested to validate the system. The concentration of each metal in the serum sample was back calculated according to standard curve to reflect the metal concentrations in original samples.

### 2.7. Statistical Analysis

All experiments were repeated as triplicates and data were presented in respective graphs as averaged values with standard deviation as error bars (*n* = 3) unless stated otherwise. OriginPro 2018b and GraphPad were used to analyze the data. All obtained fluorescent spectra were smoothed using adjacent averaging with 10 points of window and subsequently normalized to provide graph plots for comparison. BRET signals were analyzed with the equations in Table S3. To quantify the fluorescence intensity in the POC imaging, we used Fiji ImageJ [93] to split the channels for the picture and measured the mean intensity of blue and green channels in each well area. Then, the BRET ratio was calculated by dividing the mean intensity from the blue channel by the mean intensity from the green channel.

## 3. Results and Discussion

### 3.1. Design of a DNAzyme–BRET Sensor

To design the DNAzyme–BRET sensor, we used luciferase that has been fused with streptavidin (SA–luciferase) as the BRET donor. We then conjugated such SA–luciferase with 8–17 DNAzyme by a host–guest interaction between the streptavidin on the fusion protein and a biotin covalently alached to the 3′-end of the enzyme strand (E) of the DNAzyme construct (Figure 1a). In addition, a compatible fluorophore, which has an excitation spectrum that overlaps with the emission spectrum of the luciferase and thus can be excited by the bioluminescence, was conjugated to the 5′-end of substrate strand (S) as a BRET acceptor. When the DNAzyme probe was assembled, the proximity between luciferase and Cy3 fluorophore permiled BRET when coelenterazine, the substrate for luciferase, was introduced. In the presence of Zn^2+^, the DNAzyme cleaved the S strand into two shorter products, which have lower melting temperatures (T_M_) between the E strand and the S strand, and thus dehybridized these two strands in ambient conditions. As a result of this dehybridization, the fluorophore moved away from the luciferase and thus reduced the BRET efficiency, due to the increased distance between luciferase and the fluorophore. The BRET efficiency was correlated to the target concentration according to the above principle. The BRET efficiency was calculated by a ratiometric comparison between the fluorescence intensity generated by the donor luciferase and the acceptor fluorophore (BRET ratio _(fluorophore acceptor/luciferase)_).

Because a close distance between the emission peaks of the acceptor fluorophore and the luciferase generates a high background [94], we took the emission peak in addition to the excitation peak of acceptor fluorophore into consideration when choosing the fluorophore. As a result, we chose Cy3 fluorophore with an emission peak around 560 nm, which is slightly apart from the 480 nm emission peak of the SA–luciferase, instead of another fluorophore with an excitation peak around 480 nm to reduce the background while still allowing a relatively high efficiency of BRET from the SA–luciferase. Thus, we used the BRET ratio at 560 nm/480 nm (BRET ratio (560_nm/480nm)_) to evaluate the BRET efficiency, deriving from the peak emissions of luciferase (480 nm) and Cy3 (560 nm), respectively. To visualize and compare the changes of BRET ratio when adding Zn^2+^ or other analytes, we introduced a term of net BRET signal, which shows the percentage of differences between the BRET ratios (560_nm/480nm)_ in the absence (R_0_) and the presence (R) of Zn^2+^ over R0 under the same condition ([(R_0_−R)/R_0_]x100, see Table S3) [95].

### 3.2. Optimization and Demonstration of the BRET-DNAzyme Sensor

To assemble the DNAzyme probe, we annealed the S and E strands in PBS buffer by heating their mixture to 95 °C and then cooling them down to room temperature. We then added the SA–luciferase so the luciferase can bind to the biotinylated DNAzyme. To evaluate the DNAzyme probe assembly and performance in response to Zn^2+^, we used native polyacrylamide gel electrophoresis (PAGE) to monitor the hybridization between the DNA strands and the dissociation of the substrate strand from the DNAzyme’s strand in the presence of Zn^2+^. As shown in Figure S1a, upon annealing the E and S strands, the band shifted up in the gel, suggesting the formation of a DNAzyme probe. In the presence of Zn^2+^, a new band appeared with a lower molecular weight that matched the size of a synthesized cleaved product, which indicates the cleavage and dissociation of the strands.

To generate a bright fluorescence signal, we tested different concentrations of coelenterazine, and observed an increase in fluorescence intensity with the increased concentration of the substrate coelenterazine until the fluorescence intensity reached the peak with around 100 μM coelenterazine (Figure S1b). Thus, we chose 100 μM coelenterazine for later experiments. Since the luminescence generated between luciferase and its substrate was transient and faded quickly, we measured the fluorescence at 30 seconds after adding in the coelenterazine to make the detection consistent amongst groups.

We further demonstrated the DNAzyme–BRET sensor by comparing the fluorescence spectra in the absence or presence of Zn^2+^ using a fluorometer. As shown in Figure S1c, in the absence of Zn^2+^, a fluorescence signal around 480 nm alributable to the bioluminescence generated by the luciferase was observed. In addition, a small shoulder around 560 nm appeared, indicating that an energy transfer from luciferase to Cy3 occurred. When introducing 50 μM Zn^2+^ in 40 μL PBS buffer as a test sample and mixing the test sample with 120 μL of the BRET sensor solution, we observed a fluorescence intensity increase around 480 nm and a fluorescence intensity decrease around 560 nm followed by the addition of 40 μL coelenterazine solution. These changes in fluorescence intensity indicated a reduction in BRET efficiency due to Zn^2+^-dependent cleavage of the S strand by the E strand and a dissociation of the cleaved S strand fragments from the E strand, as their T_M_ are lowered after cleavage. After the dissociation, the distance between the luciferase and Cy3 was increased, resulting in less energy being transferred due to inhibited BRET, as illustrated in Figure 1a. Importantly, to prepare for applications in serum samples and establish a more straightforward calculation to reflect the Zn^2+^ concentrations in undiluted serum sample, we converted all the Zn^2+^ concentrations in this study into the concentration in the undiluted sample.

To optimize the BRET sensor’s performance and achieve a higher BRET ratio for sensing, we considered the potential interference between the conjugated luciferase and DNAzyme catalytic core. To minimize such an interference, different locations of the Cy3 fluorophore and different spacers between luciferase and the S strand were tested. We found that placing Cy3 at 5′ end of the DNAzyme, which is closer to luciferase, and having no spacer between luciferase and the S strand displayed the highest BRET ratio (560_nm/480nm)_ (Figure S2). When we increased the distance between Cy3 and luciferase by increasing the length of the spacer, the BRET ratio decreased (Figure S2), suggesting that the closer distance between luciferase and Cy3 is important for the energy transfer and can overcome the potential steric hindrance between the modifications and DNA strands.

To further optimize the BRET sensors, we have studied several conditions including annealing methods, the ratio between the S and E strands, and the ratio between the DNAzyme probes and SA–luciferase. We first tested the hybridization methods in annealing the E and S strands to verify the impact of DNA modifications (i.e., biotinylation and Cy3 conjugation) under different hybridization approaches on the subsequent BRET. As shown in Figure S3, similar BRET ratios (560_nm/480nm)_ were observed regardless of whether the annealing of the E and S strands were carried out slowly at room temperature, quicker at 4 °C, or quickest at −20 °C, suggesting that the cooling rate during the annealing between DNAzyme strands does not have significant influence on the formation of the DNAzyme construct. To ensure a low background but efficient energy transfer between the luciferase donor and the Cy3 acceptor, the stoichiometry between E and S strands must be alluded to ensure all luciferases are paired with a Cy3 fluorophore before subsequent experiments. As shown in Figure S4, the BRET ratio (560_nm/480nm)_ increased when the E:S ratio increased from 1:0 to 1:1, where a plateau was observed thereafter. This observation matched the typical observation that DNA hybridization happens at 1:1 stoichiometry. We have also tested the ratio between the DNAzyme probe and SA–luciferase. We observed a significant increase in the BRET ratio (560_nm/480nm)_ with DNAzyme probe: SA–luciferase ratio from 1:0 to 1:0.25 (Figure S5). A higher amount of luciferase did not further increase the BRET ratio (560_nm/480nm)_. Interestingly, even with SA–luciferase as lille as ¼ to that of the DNAzyme probe, the BRET ratio (560_nm/48 nm)_ was still very high and showed a significant difference between other groups with a higher amount of SA–luciferase. This ratio matched the previous report that streptavidin is a tetramer that has the potential to bind to four biotins, which also indicates that SA–luciferase can be conjugated to the DNAzyme probe as long as there is biotin available. Thus, this observation also suggests the possibility that the bioluminescence generated by one luciferase protein could be enough for transferring the energy to excite four Cy3 fluorophores. This is probably due to the exceptionally high association constant between streptavidin and biotin at about 1015 [96], suggesting that the fusion protein of streptavidin and luciferase did not weaken its affinity towards biotinylated DNA.

**Figure 2.**
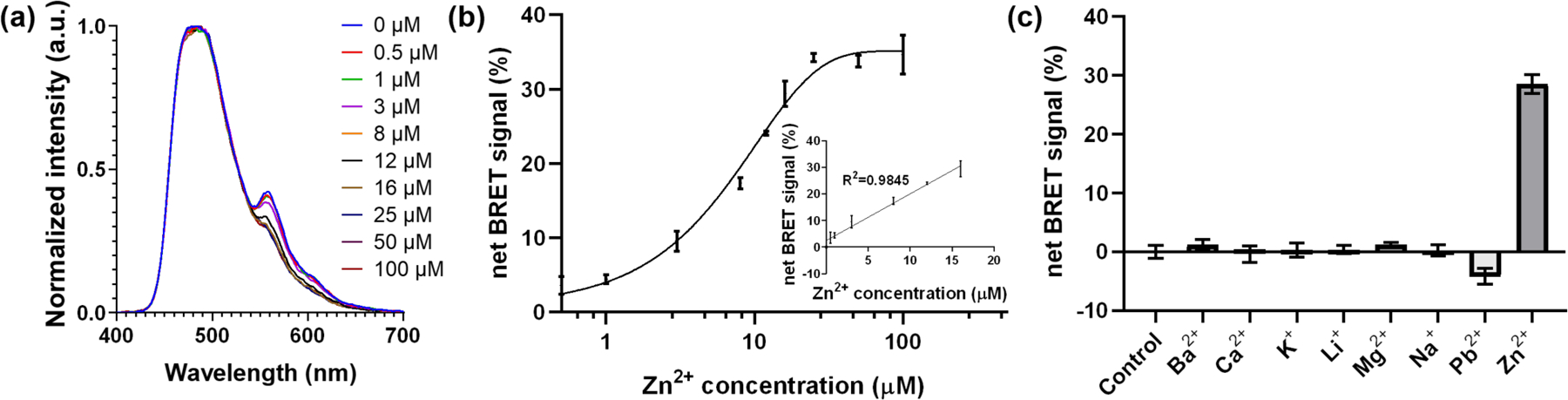
Sensitivity and selectivity assays of the BRET sensor. (**a**) Selected fluorescence spectra of the sensor upon incubation with increasing concentration of Zn^2+^ ions; (**b**) net BRET signal (%) with increasing concentration of Zn^2+^. The inset presents the linear range with up to 16 μM of Zn^2+^ in the sample. (**b**) Net BRET signal (%) of the DNAzyme–BRET sensors in the presence of 25 μM of different metal ions in PBS (pH 7.4).

Using the above sensor design and conditions, we then investigated the detection range of this BRET sensor towards Zn^2+^. We mixed 50 μL test samples, which contained Zn^2+^ concentrations from 0 to 25 μM (Figure 2a,b), with 150 μL of BRET sensor solution and detected the BRET signals right after adding 50 μL of coelenterazine solution to the mixture. We observed a correlated net BRET signal change with a linear range between 0.5 and 16 μM of Zn^2+^ (Figure 2b, inset). Based on the linear regression for this linear range, we calculated the limit of detection (LOD) based on the standard deviation of the net BRET signal response (Sy, which equals to 1.530 in this curve) of the curve and the slope of the calibration curve (S, which equals to 1.758 in this curve) according to the equation of LOD = 3.3 x (Sy/S) [97] and obtained a LOD for Zn^2+^ of 2.87 μM. This limit of detection is close to the detection limit of 1 μM as the commercially available Zn^2+^ indicator FluoZin^™^−3, and the detection limit of 1.5 μM of the small molecule sensor 5-Br-PADAP [33].

Next, we investigated the selectivity of the sensor by challenging it with different mono- and divalent metal ions. Previous studies have shown the high selectivity of the 817 DNAzyme for Zn^2+^ over other metal ions, including potassium (K^+^), lithium (Li^+^), sodium (Na^+^), barium (Ba^2+^), calcium (Ca^2+^), magnesium (Mg^2+^), and lead (Pb^2+^). We confirmed this observation with metals at the concentration of 25 μM in the sample, which was the concentration that the net BRET signal reached plateau in Figure 2b. Among all the metal ions we tested, only the Zn^2+^ showed a significant increase in net BRET signal (Figures 2c and S6). Although some of the metal ions, such as K^+^, Na^+^, Ca^2+^, and Mg^2+^, have higher physiological concentrations than 25 μM (see Table S4), and may interact with the DNAzyme at their physiological concentrations, the DNAzyme activity with 10 μM Zn^2+^ is around 1.5 to 2-fold over its activity with 1 mM of these metal ions and 10 μM of Fe^2+^ [98]. On the other hand, trivalent metal ions, such as Fe^3+^ have minimal interaction with the 8–17 DNAzyme [98], thus will not likely to interference with the detection. In comparison, the commercially available Zn^2+^ sensor FluoZin^™^−3 is less selective, which showed around 1.6-fold activity with 1 μM Zn^2+^ over 100 μM of Ca^2+^ and Mg^2+^, and has minimal selectivity over Fe^2+^ and Hg^2+^ [99,100]; while 5-Br-PADAP is also reactive with Cu^2+^ and achieved a detection limit of 3.80 × 10^–2^ ng/mL with Cu^2+^ [40].

### 3.3. Zn^2+^ Detection in Human Serum with Smartphone

**Figure 3.**
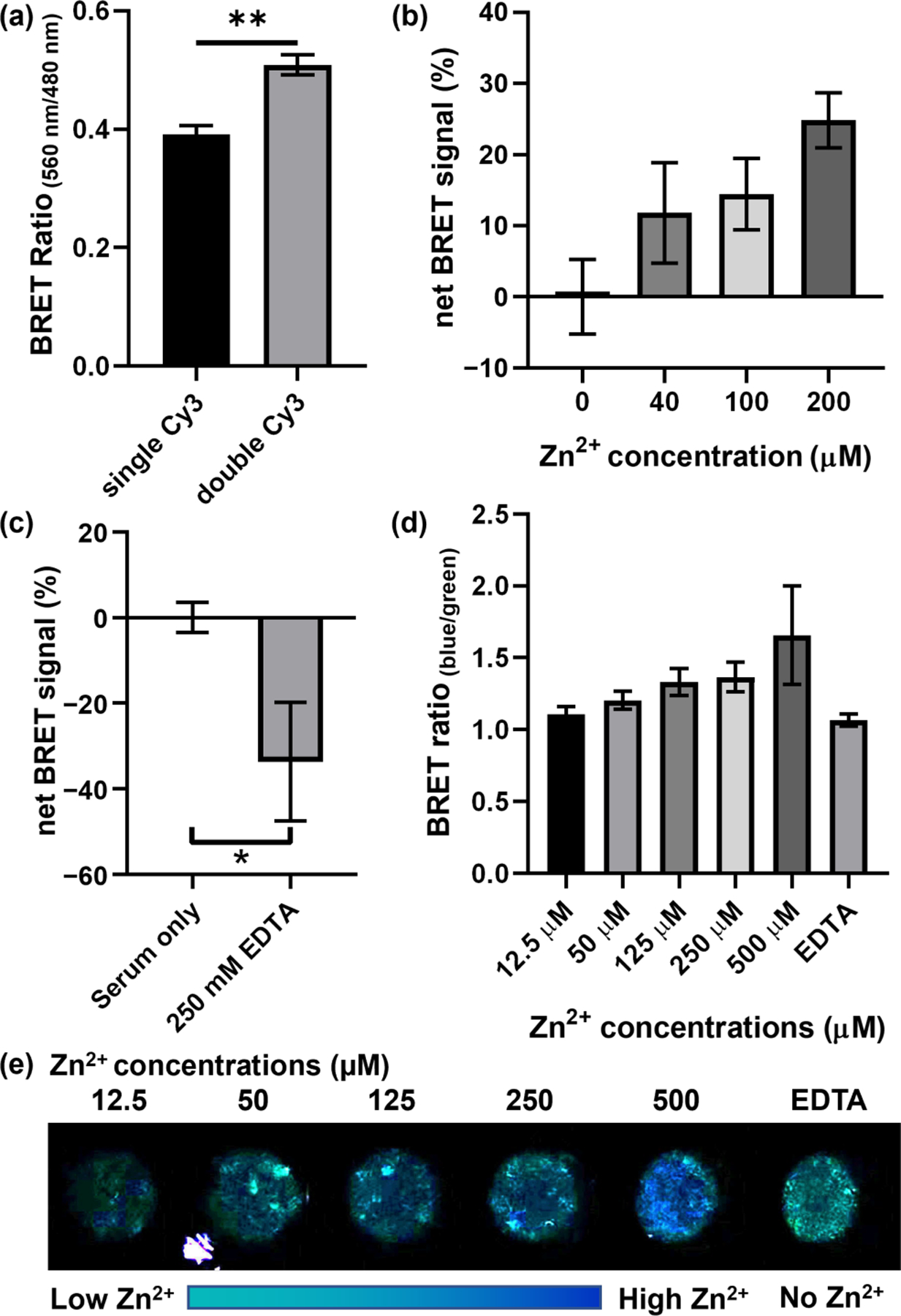
Detection of Zn^2+^ in human serum. (**a**) Comparison of BRET ratios (560_nm/480nm)_ between sensors with single Cy3 acceptor and the dual-Cy3 acceptors; (**b**) net BRET signal reveals Zn^2+^ concentration change in 1:1 diluted human serum; (**c**) net BRET signal decreases when quenching metal ions with EDTA; (**d**) Image J based quantification of the POC imaging reflects that net BRET ratio signal increases when Zn^2+^ concentration increases; and (**e**) POC imaging of Zn^2+^ concentration in 1:1 diluted human serum with smartphone camera. Three biological repeats were tested, and the error bars were generated based on independent experiments. * *p* < 0.05; ** *p* < 0.01, with student-t test.

Despite the promising results of detecting Zn^2+^ both quantitatively and qualitatively, the BRET signal’s color change obtained using the single-acceptor BRET sensor was not strong enough to be visualized using a smartphone due to the emission from Cy3 being weak and overwhelmed by the luciferase’s bioluminescence. This low-energy transfer efficiency is also reflected in the small signal change displayed in Figure 2a. To overcome this limitation, we were inspired by the design of the DNAzyme catalytic beacon with dual quenchers, which showed beler energy transfer and quenching of fluorescence of the fluorophore when using both inter- and intra-molecule quenchers instead of one intermolecular quencher [57], and added a second Cy3 acceptor to increase the energy transfer (Figure 1b). With this modification, we were able to observe a higher BRET ratio (560_nm/480nm)_ with two Cy3 acceptors instead of one single acceptor without Zn^2+^ (Figure 3a). Since we observed earlier in Figure S2 that a shorter distance between the Cy3 and luciferase in the single Cy3 acceptor design showed a higher BRET ratio (560_nm/480nm)_, we hypothesized that a shorter distance between the second Cy3 acceptor and the luciferase donor in the dual-Cy3 acceptor design can further increase the BRET ratio (560_nm/480nm)_. To test this hypothesis, we compared the BRET ratio (560_nm/480nm)_ between the DNA probe with a 20-base or 26-base S strand. We observed a higher BRET ratio (560_nm/480nm)_ in the probe that has a shorter S strand and thus a shorter distance between the donor/acceptor pairs (Figure S7). This result suggests that the distance between the Cy3 and the luciferase is critical for BRET. We did not investigate an S strand that is shorter than 20 bases because the melting temperature between the S and E strands would be too low to stay completely hybridized during the storage and applications. Similar to FRET, the energy transfer can vary with the distance between the donor and acceptor at sixth power of their distance [101,102]. Specifically, the shorter distance may help to increase the energy transfer between the luciferase and its intra-molecule acceptor on the other end of the substrate strand. Thus, we focused on the two-Cy3 acceptor system that with only a 20-base S strand for our further studies.

To validate if this new BRET sensor can achieve detection of Zn^2+^ in serum samples, we spiked different concentrations of Zn^2+^ into serum samples. The spiked samples were 1:1 diluted with PBS–zinc buffer to generate a 40 μL sample solution with different concentrations of Zn^2+^, which serve as a proof-of-concept demonstration of detecting Zn^2+^ in a 40 μL sample. This 1/2 diluted sample solution was then mixed with 120 μL of the BRET sensor solution. After adding 40 μL of coelenterazine, we observed an increase in net BRET signals, which correlated with the concentrations of Zn^2+^ in the sample solution (Figure 3b). To validate this observation and evaluate the performance of our sensors in serum samples, we used ICP-MS to measure a broad spectrum of metal ions in a serum sample and 10% serum samples spiked with different concentrations of Zn^2+^. According to the results, there were around 26.3 μM Zn, 148.0 mM Na, 149.7 μM Mg, 2.2 mM Ca, 37.8 μM Fe, 14.1 μM Cu, and 0.5 μM Pb in the human serum sample we tested. To mimic the conditions that we used for testing the BRET sensors in human serum environment, we also performed ICP-MS on metal ions in 10% serum spiked with 40, 100, and 200 μM of Zn^2+^. As shown Table S4, the amount of Zn increased with increasing concentrations of Zn^2+^ that we added, while the amount of other metal ions did not show significant changes between different spiked groups. Therefore, the increase in the net BRET signal we observed was correlated with the elevation of Zn^2+^ concentration in the serum sample, which indicates that our sensor was able to detect the presence of different concentrations of Zn^2+^ in the serum environment. However, it is difficult to find a direct quantitative correlation between the concentrations obtained from ICP-MS and those from the BRET sensors, because the former measure the total amount of metal ions in all oxidation states, while the laler measure labile metal ions in a specific oxidation state. Additionally, we have also observed a 33.6% decrease in the net BRET signal when introducing 250 mM EDTA in the sample solution to chelate divalent metal ions (Figure 3c). To further validate the performance of our sensors, we applied our DNAzyme sensor in 40 μL 1:1 diluted fetal bovine serum (FBS) or 80 μL undiluted FBS and observed a similar correlation between net BRET signal and Zn^2+^ concentrations (Figure S8a–c) in both samples. Therefore, our sensor achieved semi-quantitative Zn^2+^ detection in a variety of serum samples with different dilution factors. More importantly, the undiluted FBS showed a 15% higher BRET signal change when compared with 1:1 diluted FBS, suggesting that a higher concentration of Zn^2+^ was detected in less diluted serum samples (Figure S8d).

One of the advantages of the BRET sensor is that it does not require laser excitation, thus it does not require sophisticated equipment for sensing. To test the ability of using the sensor for a quick detection of Zn^2+^ in serum samples, we imaged the BRET signaling in the 1:1 diluted human serum with a smartphone camera (Figure 3d–e). In the presence of 250 mM EDTA in the serum sample, the image showed a green color, which could be generated by a combined signal from Cy3 and luciferase and indicates Cy3 was excited by the bioluminescence. This inspiring result is consistent with the data collected using the fluorometer, which showed a lower net BRET signal with the addition of EDTA (Figure 3c). When the same sample is spiked with 500 μM Zn^2+^, a blue color was observed, which indicates a higher bioluminescence instead of energy transfer and exciting Cy3 fluorophores. The samples spiked with lower Zn^2+^ concentrations showed an intermediate color between the blue color of the 500 μM Zn^2+^ spiked sample and the green color of the EDTA spiked sample, suggesting that the color of the smartphone pictures can be used to reveal the relative amount of Zn^2+^ in real samples. To further evaluate how well the smartphone imaging correlates to serum Zn^2+^ levels and generate a quantifiable result, we used ImageJ to quantify the average fluorescence intensity in the blue and green channels of each sample and observed an increase in the BRET ratio (_blue/green)_ correlated to different concentrations of spiked in Zn^2+^ (Figure 3d). These results suggest that our sensor can indicate Zn^2+^ concentration with a smartphone-based quick detection. Since the catalytic core and the signal output element are separate in our BRET–DNAzyme sensor, this sensor design would be generalizable and adaptable for sensing other metal ions in the future by changing the 8–17 DNAzyme into another DNAzyme that is specific to the target metal ion. Therefore, this sensing system has great potential to be widely used for detecting metal ions in health and disease conditions.

## 4. Conclusions

In summary, we have developed and demonstrated DNAzyme-based BRET sensors for measuring metal ions in serum samples. By incorporating luciferase modified with streptavidin, we have not only introduced BRET to the DNAzyme system, but also achieved a ratiometric measurement of Zn^2+^ in human serum with a smartphone. This design can be generalized and applied to other DNAzyme systems as well as for monitoring other metal ions. Ultimately, this work should serve as a promising foundation to support further efforts for the development of affordable and portable metal ion POC detectors by eliminating the need of a light excitation source.

## Supplementary Material

SI

## Data Availability

The data presented in this study are available in this article and related supplementary material.
