# Supplementary material for: Ratiometric Detection of Zn2+ Using DNAzyme-Based Bioluminescence Resonance Energy Transfer Sensors
Source: Chemistry (Basel). Author manuscript; Available in PMC 2024 Feb 18. (PMC10874629; doi:10.3390/chemistry5030119)
Supplement: SI [file NIHMS1963578-supplement-SI.docx]

Supporting Information for

Ratiometric Detection of Zn^2+^ using DNAzyme-based Bioluminescence Resonance Energy Transfer Sensors

Yuting Wu ^1,2^^,^†, Whitney Lewis ^1,2,^†, Jing Luen Wai ^2,3,^†, Mengyi Xiong ^2,4^, Jiao Zheng ^2^, Zhenglin Yang ^1,5^, Chloe Gordon^1^, Ying Lu ^1^, Siu Yee New ^3^, Xiao-Bing Zhang ^4^, and Yi Lu ^1,2,5,^*

^1^ Department of Chemistry, University of Texas at Austin, Austin, Texas 78712, United States

^2^ Department of Chemistry, University of Illinois at Urbana-Champaign, Urbana, Illinois 61801, United States

^3^ School of Pharmacy, Faculty of Science and Engineering, University of Nottingham Malaysia, Semenyih, Selangor 43500, Malaysia

^4^ Molecular Science and Biomedicine Laboratory, State Key Laboratory of Chemo/Biosensing and Chemometrics, College of Chemistry and Chemical Engineering, Hunan University, Changsha, Hunan 410082, China

^5^ Department of Biochemistry, University of Illinois at Urbana-Champaign; Urbana, Illinois 61801, United States

***** Correspondence: [yi.lu@utexas.edu](mailto:yi.lu@utexas.edu); † Co-first authors

Table of Contents

[Table of Contents 2](#_Toc141981080)

[Supplemental Figures 3](#_Toc141981081)

**[Figure S1.](#_Toc141981082)***[In vitro](#_Toc141981082)* [visualization of the BRET system. 3](#_Toc141981082)

**[Figure S2.](#_Toc141981083)** [Optimization of the spacer in the single BRET systems. 4](#_Toc141981083)

**[Figure S3.](#_Toc141981084)** [Comparison of cool down techniques for annealing the DNAzyme strands. 4](#_Toc141981084)

**[Figure S4](#_Toc141981085)**[. Titration of E and S strand ratio. 5](#_Toc141981085)

**[Figure S5.](#_Toc141981086)** [Titration of different ratios between DNAzyme probe (ES) and SA-luciferase. 5](#_Toc141981086)

**[Figure S6.](#_Toc141981087)** [Fluorescence spectra for selectivity assay of the sensor. 6](#_Toc141981087)

**[Figure S7.](#_Toc141981088)** [The length of S strand influence on the BRET intensity. 6](#_Toc141981088)

**[Figure S8.](#_Toc141981089)** [Zn](#_Toc141981089)^[2+](#_Toc141981089)^ [sensing in fetal bovine serum (FBS). 7](#_Toc141981089)

[Supplemental Tables 8](#_Toc141981090)

**[Table S1.](#_Toc141981091)** [A comparison of advantages and limitations between techniques for Zn](#_Toc141981091)^[2+](#_Toc141981091)^ [detection. 8](#_Toc141981091)

**[Table S2.](#_Toc141981092)** [DNA sequences used in this study. 8](#_Toc141981092)

**[Table S3.](#_Toc141981093)** [Equations and definitions used for analyzing BRET signals. 9](#_Toc141981093)

**[Table S4.](#_Toc141981094)** [Metal concentrations in serum samples.* 9](#_Toc141981094)


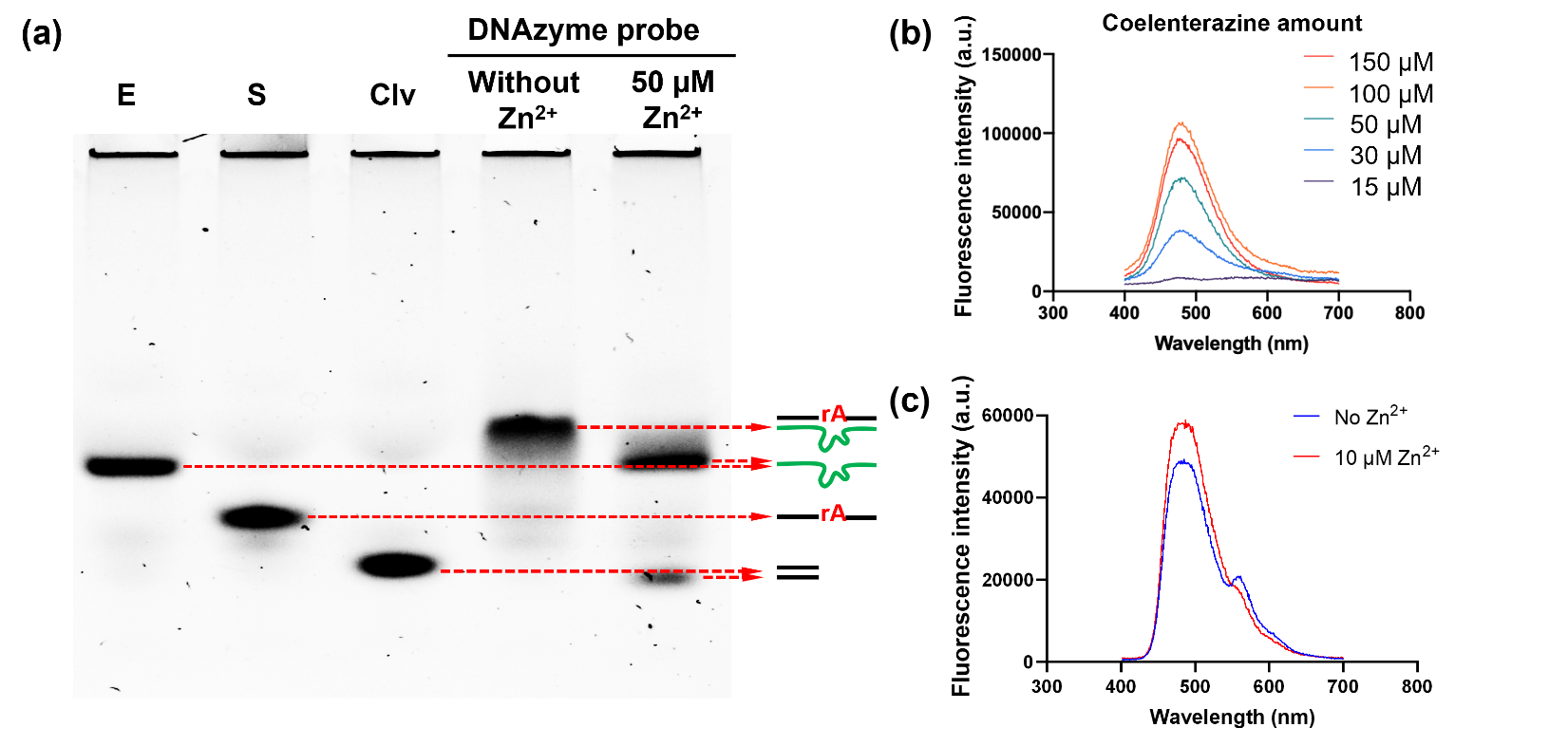
Supplemental Figures

**Figure S1.** *In vitro* visualization of the BRET system.

(**a**) 12% native PAGE visualizing the formation of the double stranded DNAzyme probe and its activity in the presence of Zn^2+^ ions. The gel was stained with SYBR Gold for visualization. Lane 1 Cy3 labelled E strand; Lane 2: Cy3 and biotin dual modified S strand; Lane 3: Synthesized cleaved product with Cy3 labeling; Lane 4: DNAzyme probe without Zn^2+^; Lane 4: DNAzyme probe with 50 µM Zn^2+^ in sample (40 µl, 1/5 of the total reaction volume). Red lines point to the scheme of potential structures of each size of bands shown on the right side of the gel. (**b**) Spectra of bioluminescence generated at 30 seconds after introducing different concentration of coelenterazine. (**c**) Spectra of the DNAzyme-BRET sensor without or with 50 µM Zn^2+^ in sample.

**
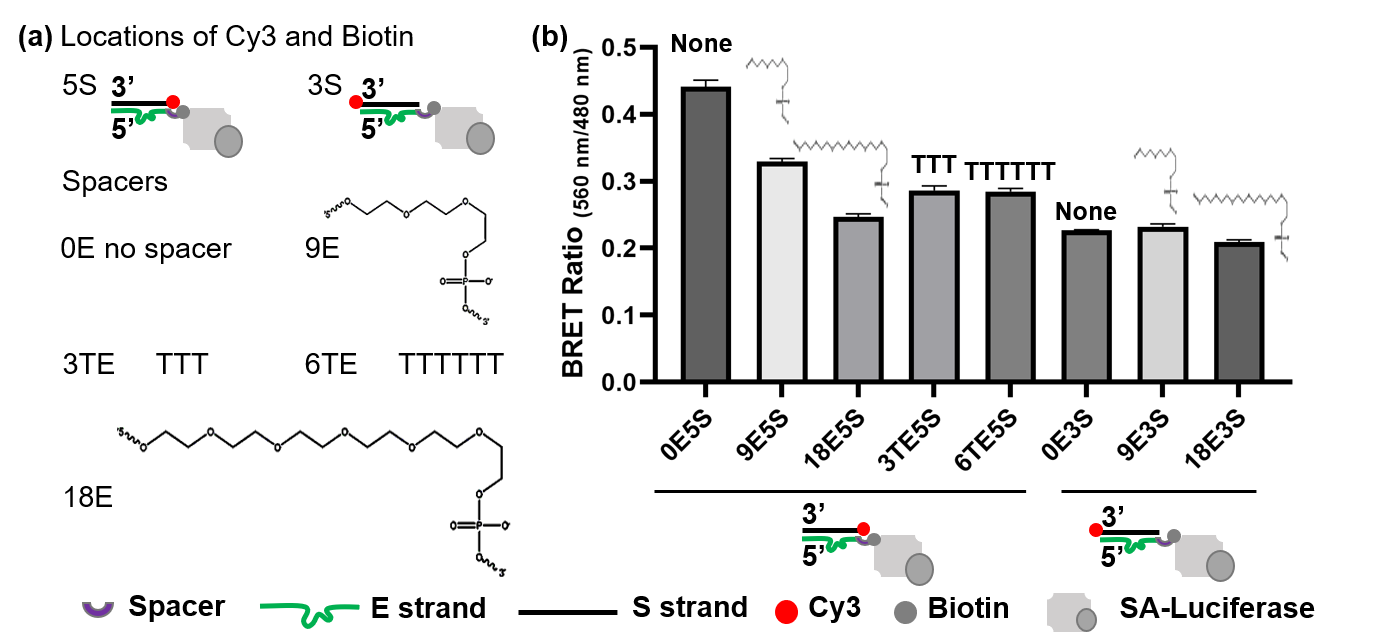
**

**Figure S2.** Optimization of the spacer in the single BRET systems.

BRET ratio comparison between different positions of the Cy3 acceptor and different lengths of linkers between the DNAzyme and biotin modification. (**a**) The information of different linkers: 3S, Cy3 located at the 3’ terminal of the S strand; 5S, Cy3 located at the 5’ terminal of the S strand. 0E, 9E, 18E, 3TE, 6TE, represent different spacers between the end of the E strand and the biotin modification for SA-luciferase conjugation. 0E, no spacer; 9E, triethylene glycol spacer; 18E, 18-atom hexa-ethyleneglycol spacer; 3TE, trithymidylate spacer; 6TE, hexathymidylate spacer. (**b**) The BRET ratio (560 nm/480 nm) demonstrates the comparison of BRET efficiency between the sensors with different linkers and Cy3 locations.


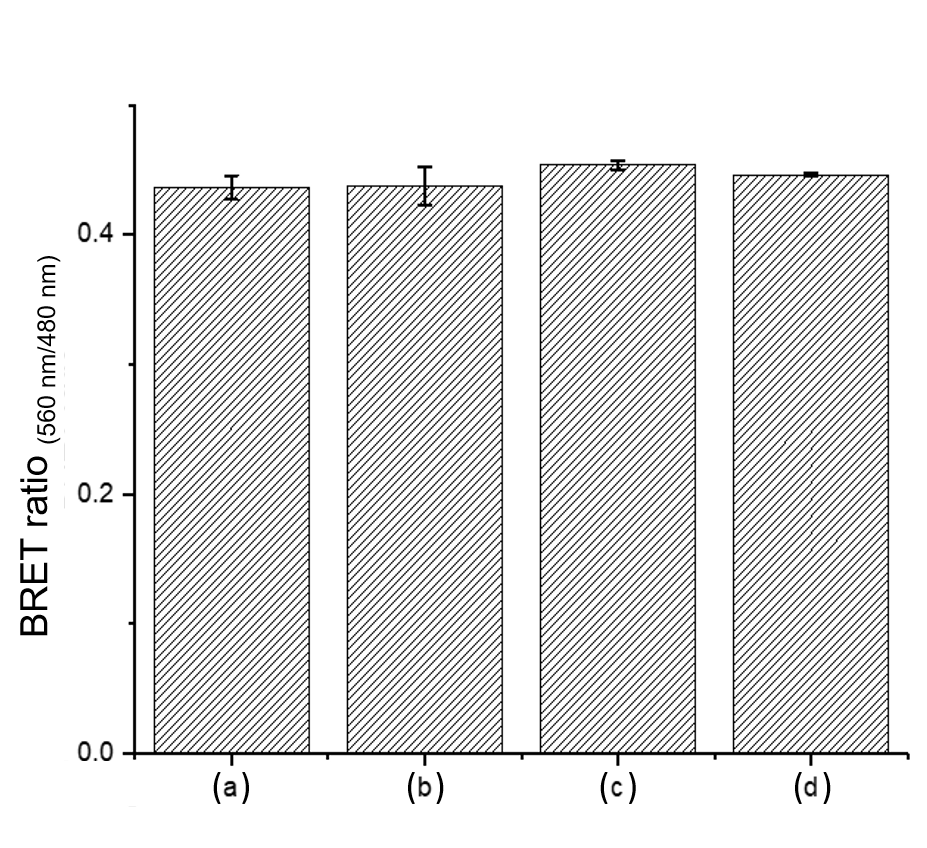
**Figure S3.** Comparison of cool down techniques for annealing the DNAzyme strands.

After heating up to 95°C for 5 minutes, the DNAzyme strands were cooldown with (**a**) room temperature (60 minutes); (**b**) 4°C fridge (60 minutes); (**c**) 4°C fridge (45 minutes) then -20°C freezer (15 minutes); (**d**) room temperature (30 minutes) then 4°C fridge (30 minutes).

**
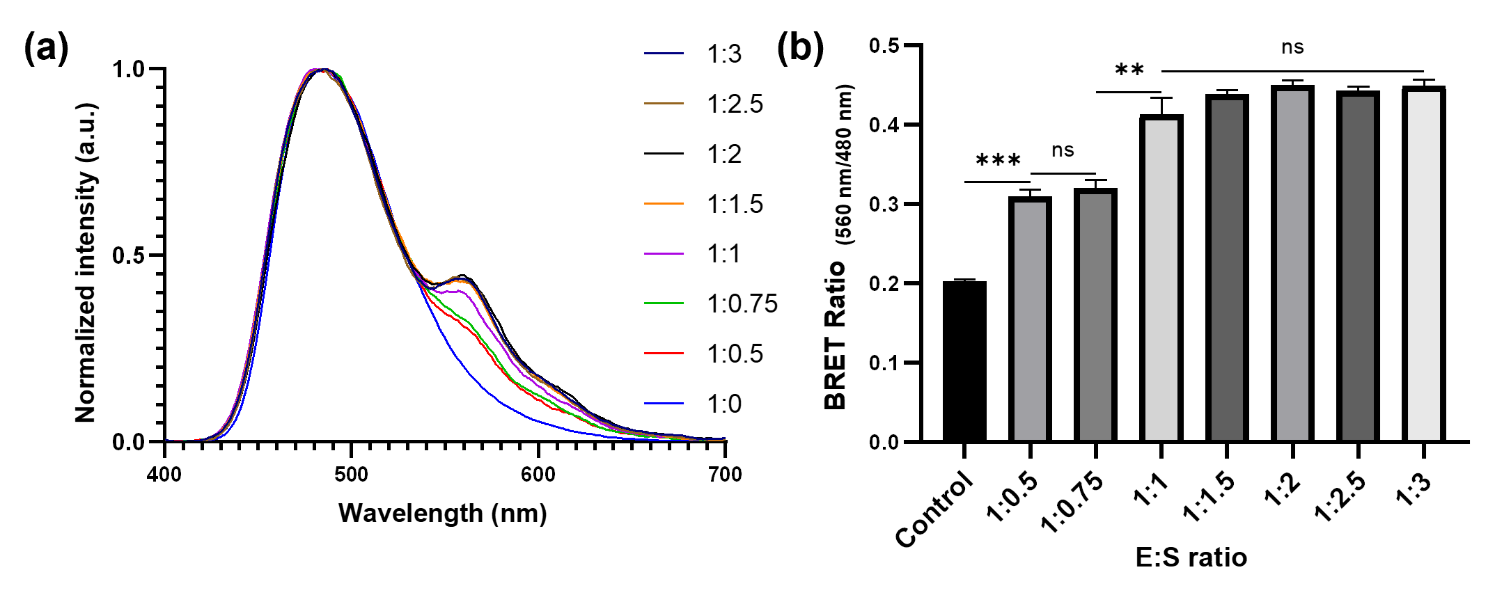
**

**Figure S4**. Titration of E and S strand ratio.

(**a**) Spectra of the different ratios between E and S strands when forming the DNAzyme probe. (**b**) BRET ratio (560 nm/480 nm) show the titration of influence from different ratios between E and S strands when forming the DNAzyme probe. Unpaired student-t tests were used for statistical analysis. **, *p* <0.01; ***, *p* <0.001; ns, *p* > 0.05.

**Figure S5.** Titration of different ratios between DNAzyme probe (ES) and SA-luciferase.

The BRET ratio from the group of 1:0 DNAzyme probe:SA-luciferase ratio is significantly lower than other groups (*p* = 0.0002 between the 1:0 and 1:0.25 group). There was no significant difference between other groups according to unpaired student-t test (*p* > 0.05).

**Figure S6.** Fluorescence spectra for selectivity assay of the sensor.

40 µL test samples in PBS buffer were incubated with 25 µM of various metal ions.

**

**

**Figure S7.** The length of S strand influence on the BRET intensity.

Fluorescence spectra showed a higher emission pick with 20 bases S strand (b20) than 26 bases S strand (b26), indicating that that A 20 bases S strand has a higher BRET than a 26 bases S strand.

**
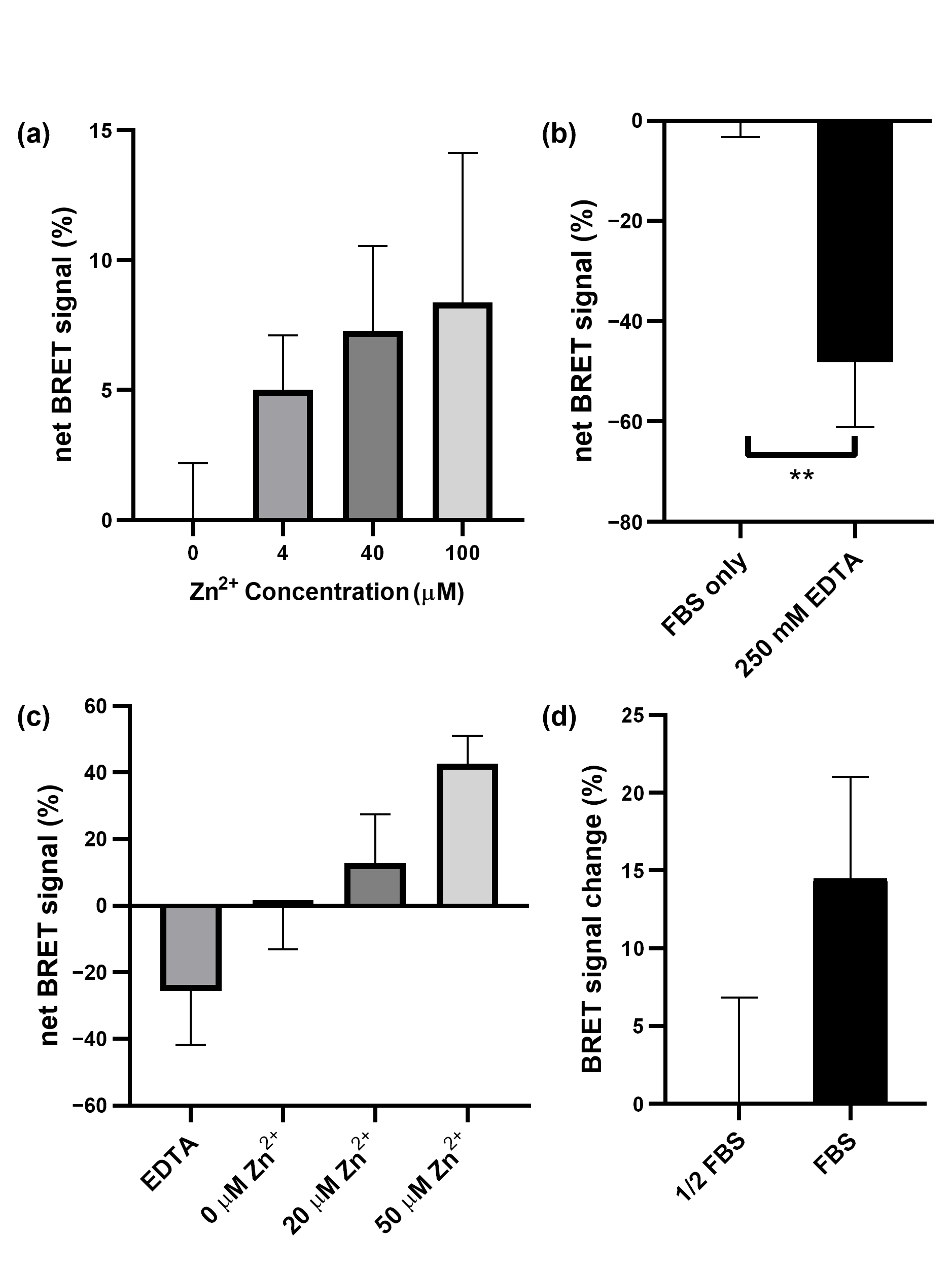
**

**Figure S8.** Zn^2+^ sensing in fetal bovine serum (FBS).

(**a**) Zn^2+^ sensing in 1:2 diluted FBS; (**b**) adding 250 mM EDTA in the 1:2 diluted FBS increased the BRET ratio; (**c**) Zn^2+^ sensing in undiluted FBS; (**d**) a comparison of BRET signal change between different dilutions of FBS.

Supplemental Tables

**Table S1.** A comparison of advantages and limitations between techniques for Zn^2+^ detection.

| **Techniques** | **Example** | **Advantages** | **Limitations** |
| --- | --- | --- | --- |
| Spectrometry technologies | Inductively coupled plasma mass spectrometry [28–32] | Available for most  metal ions | Need sophisticated instruments and well-trained technicians,  Not suitable for POC detection |
| Fluorescence-based sensors | Small molecule sensors [33–38] | Can be used in  POC settings | Limited selectivity,  Not generalizable for other metal ions |
|  | Protein based sensors [91] |  |  |
|  | DNAzyme based sensors [43,70–77] | Highly selective and generalizable | Laser excitation requires specific equipment which generates high autofluorescence background  and photobleaching |
| Chemiluminescence resonance energy transfer sensors | DNAzyme based CRET sensor [85] | Do not require  excitation light source,  Rapid and portable  metal detection | Rely on H_2_O_2_,  and difficult to apply in  various biological samples |
| Bioluminescence resonance energy transfer sensors | Small molecule sensors [89] | Biocompatible,  Low background interference and minimal photobleaching | Limited selectivity,  Not generalizable for other metal ions |
|  | Protein based sensors [88] |  |  |
|  | DNAzyme based sensors  (This work) | All advantages  listed above | Short light emission time,  need to detect immediately |

**Table S2.** DNA sequences used in this study.

| Name | Sequence (5'-3') |
| --- | --- |
| 8-17 0E | TCCATCTCTTCTCCGAGCCGGTCGAAATAGTGAGT/3Bio/ |
| 8-17 3TE | TCCATCTCTTCTCCGAGCCGGTCGAAATAGTGAGTTTT/3Bio/ |
| 8-17 6TE | TCCATCTCTTCTCCGAGCCGGTCGAAATAGTGAGTTTTTTT/3Bio/ |
| 8-17 9E | TCCATCTCTTCTCCGAGCCGGTCGAAATAGTGAGT/iSp9//3Bio/ |
| 8-17 18E | TCCATCTCTTCTCCGAGCCGGTCGAAATAGTGAGT/iSp18//3Bio/ |
| 8-17 S 5’Cy3 | /5Cy3/ACTCACTATrAGGAAGAGATGGA |
| 8-17 S 3’Cy3 | ACTCACTATrAGGAAGAGATGGA/3Cy3Sp/ |
| 8-17 E 3’Cy3 | CATCTCTTCTCCGAGCCGGTCGAAATAGTGAGT/3Cy3Sp/ |
| 8-17 S 5'bio-3'Cy3 (20 bases S) | /52-Bio/ACTCACTATrAGGAAGAGATG/3Cy3Sp/ |
| 8-17 S 5'bio-3'Cy3 (26 bases S) | /52-Bio/ACTCACTATrAGGAAGAGATGGACGTG/3Cy3Sp/ |
| 8-17 E 5'Cy3 | /5Cy3/CATCTCTTCTCCGAGCCGGTCGAAATAGTGAGT |
| 8-17 S 5'Cy3-3'bio | /5Cy3/ACTCACTATrAGGAAGAGATG/3Bio/ |
| 8-17 S Cleaved 3'Cy3 | AGGAAGAGATG/3Cy3Sp/ |

**Table S3.** Equations and definitions used for analyzing BRET signals.

| Symbol | Definition or calculation for the symbol |
| --- | --- |
| BRET ratio (560 nm/480 nm) | Fluorescence intensity at 560 nm/480 nm in the normalized and smoothed spectra curve |
| R | BRET ratio (560 nm/480 nm) with different concentrations of Zn^2+^ or EDTA |
| R0 * | The averaged BRET ratio (560 nm/480 nm) in groups that did not spike in Zn^2+^ or EDTA |
| Net BRET signal [% over basal] | [(R0-R)/R0]x100 |

* For the comparison between different dilutions of serum sample, which all did not add in additional Zn^2+^, we used the averaged BRET ratio (560 nm/480 nm) in lowest diluted group to serve as R0 for calculating the BRET signal changes [% over 1/2 diluted serum] with the same equation.

**Table S4.** Metal concentrations in serum samples.*

| Metal ions | Reported amount | Human Serum | 10% Serum spiked 0 µM ZnCl_2_ | 10% Serum spiked 40 µM ZnCl_2_ | 10% Serum spiked 100 µM ZnCl_2_ | 10% Serum spiked 200 µM ZnCl_2_ |
| --- | --- | --- | --- | --- | --- | --- |
| Na | 137 mM [103] | 148.0 mM | 159.7 mM | 156.4 mM | 161.3 mM | 161.4 mM |
| Mg | 850 µM [104] | 149.7 µM | 28.0 µM | 24.6 µM | 33.2 µM | 28.5 µM |
| Ca | 2.2 mM [105] | 2.2 mM | 309.8 µM | 237.7 µM | 295.9 µM | 290.8 µM |
| Fe | 10.74 to 30.43 µM [106–109] | 37.8 µM | 21.5 µM | 18.4 µM | 24.4 µM | 20.5 µM |
| Cu | 15.9 µM [110,108] | 14.1 µM | 3.1 µM | 3.1 µM | 3.2 µM | 3.2 µM |
| **Zn** | 15 µM [110,108] | 26.3 µM | 15 µM | 44.0 µM | 115.6 µM | 222.7 µM |
| Rb | 2.8 µM [108] | 0.5 µM | 0.2 µM | 0.2 µM | 0.2 µM | 0.2 µM |

* The metal concentrations in human serum were detected with ICP-MS. All the concentrations were shown as mean value. 10% Serum were diluted with PBS (without Ca^2+^ and Mg^2+^), which mimicked the condition we used for BRET sensor-based detections, and then diluted an additional 250-fold with 2% HNO_3_ before testing. The concentration of metals in 10% serum sample represents the concentration in the PBS buffer. Other ions such as Al, Mn, Co, Ni, As, Sr, Zr, Mo, Ag, Cd, Ba, Tl, and Pb were also tested, but they were either below the limit of detection or did not show concentration differences with different dilutions of serum samples.
